# Screening of *N-*Alkyl-Cyanoacetamido Oximes as Substitutes for *N-*Hydroxysuccinimide

**DOI:** 10.1002/open.201200012

**Published:** 2012-06-11

**Authors:** Sherine N Khattab, Ramon Subirós-Funosas, Ayman El-Faham, Fernando Albericio

**Affiliations:** a Department of Chemistry, Faculty of Science, University of AlexandriaP.O. Box 246, Ibrahimia, 21321 Alexandria (Egypt) E-mail: aymanel_faham@hotmail.com; b Department of Chemistry and Molecular Pharmacology, Institute for Research in BiomedicineBarcelona Science Park, Baldiri Reixac 10, 08028 Barcelona (Spain) E-mail: albericio@irbbarcelona.org; c CIBER-BBN, Networking Centre on Bioengineering, Biomaterials and NanomedicineBarcelona Science Park, Baldiri Reixac 10, 08028 Barcelona (Spain); d Department of Chemistry, College of Science, King Saud UniversityP.O.Box 2455, 11451 Riyadh (Kingdom of Saudi Arabia); e Department of Organic Chemistry, University of BarcelonaMartí i Franqués 1–11, 08028 Barcelona (Spain); f School of Chemistry, University of KwaZulu-Natal4041 Durban (South Africa)

**Keywords:** additives, *N*-hydroxysuccinimides, oximes, peptide-bond formations, peptide syntheses

## Abstract

Peptide-bond formation is a pivotal process in the synthesis of peptide oligomers. Among the various coupling methodologies described, carbodiimides combine strong acylation potency and smooth reaction conditions, and they are commonly used in the presence of *N*-hydroxylamine additives. In recent years, acidic oxime templates, mainly ethyl 2-cyano-2-(hydroxyimino) acetate (Oxyma), have emerged as highly reactive alternatives to the classic and explosive-prone benzotriazolic additives, 1-hydroxybenzotriazole (HOBt) and 1-hydroxy-7-azabenzotriazole (HOAt). However, to achieve certain biochemical targets, less reactive species, such as *N*-hydroxysuccinimide (HOSu) esters, are often required to obtain stability under aqueous conditions. In the present study, we report on a new family of water-soluble *N*-alkyl-cyanoacetamido oximes, most of which have proven useful in the construction of active carbonates for the introduction of fluorenylmethoxycarbonyl (Fmoc) with minimal impact of dipeptide impurities. We performed a direct comparison of these new *N*-alkyl-cyanoacetamido oximes with HOSu in order to evaluate their capacity to retain optical purity and their coupling efficiency in the assembly of bulky residues.

## Introduction

**Figure 1:**
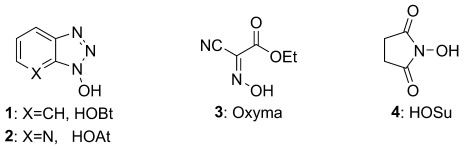
Structure of *N*-hydroxylamines currently available for use as additives to carbodiimides.

The choice of the active acylating species used to form a peptide bond is crucial in peptide synthesis.[1–4] Highly reactive moieties, such as acid halides, are suitable to acylate very poor nucleophilic amines. However, they are often not the best choice for the activation of the *N^α^-*alkoxycarbonyl derivatives of amino acids [mostly fluorenylmethoxycarbonyl (Fmoc), *tert-*butoxycarbonyl (Boc), and allyloxycarbonyl (Alloc)] because of their marked electrophilic character, which derives in a considerable loss of configuration.[5–9] In contrast to acid chlorides, the corresponding active esters offer a more balanced range of reactivity, thus, circumventing most of the side reactions caused by an excessive activation.[1, 2, 9, 10] From the extensive range of these esters, *N-*hydroxylamine esters, preferably prepared in situ by the carbodiimide approach using the corresponding *N*-hydroxylamines as additives, are possibly the most widely used.[11–13] Among these, 1-hydroxybenzotriazole (HOBt, **1**) and its derivatives, including 1-hydroxy-7-azabenzotriazole (HOAt, **2**), have prevailed over the other carbodiimide additives during recent decades as a result of their great acylation potency (Figure 1).[12, 14, 15] However, a few years ago, the explosive nature of benzotriazoles prompted the reevaluation of the strongly acidic oxime, ethyl 2-cyano-2-(hydroxyimino)acetate (Oxyma, **3**) as a potential replacement, which represented a hallmark in the field.[16, 17]

**Figure 2:**
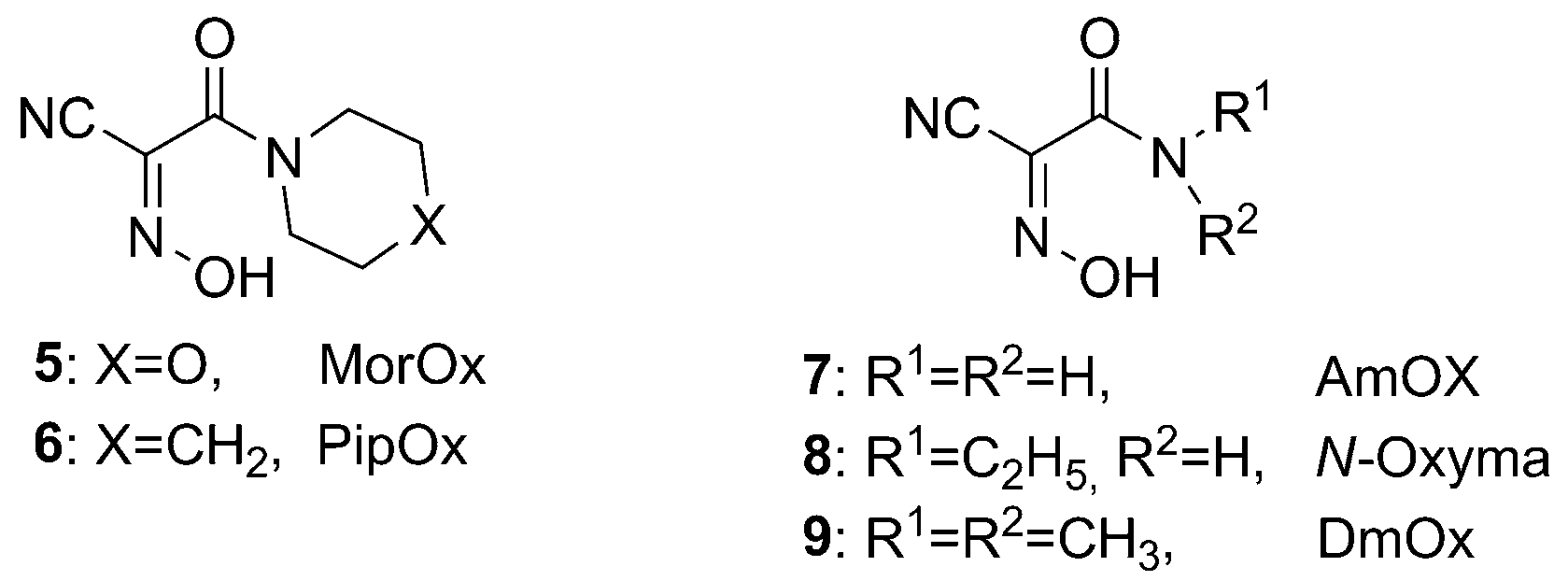
Structure of the *N*-alkyl-cyanoacetamido oximes proposed herein.

In contrast to standard peptide-bond formation, which occurs in common organic solvents, the acylation of peptides or proteins for bioconjugation purposes typically takes place in aqueous pH-buffered environments. Thus, stability rather than reactivity is the main desired feature of active esters generated in such a polar medium. In a similar manner, preformed amino acid active esters need to be stable enough for shelf storage of up to several weeks. Consequently, *N*-hydroxysuccinimide esters (HOSu, **4**) have found broad application in these scenarios as less reactive but more suitable alternatives to benzotriazoles (Figure 1).[30–31] For example, HOSu derivatives **4** have been used for the preparation of alkoxycarbonyl amino acids, thereby avoiding the undesired formation of oligomers.[20] However, the limited reactivity of HOSu active species continues to be a decisive drawback. In order to pursue an optimal balance of reactivity for stable active esters, we focused our attention on a family of promising *N-*alkyl-cyanoacetamido oximes (**5**–**8**, Figure 2). In a recent publication, Fmoc-carbonates derived from *N*-hydroxy-2-morpholino-2-oxoacetimidoyl cyanide (MorOx, **5**), *N*-hydroxy-2-oxo-2-(piperidin-1-yl) acetimidoyl cyanide (PipOx, **6**), 2-amino-*N*-hydroxy-2-oxoacetimidoyl cyanide (AmOx, **7**), and 2-(ethylamino)-*N*-hydroxy-2-oxoacetimidoyl cyanide *(N*-Oxyma, **8**) presented desirable reactivities with superior performances to Fmoc-OSu (Figure 2).[21]

Following previous research on Fmoc-oxime carbonates, the present study aimed at performing a screen of *N-*alkyl-cyanoacetamido oximes **5**–**8** as additives to carbodiimides in replacement of HOSu (**4**). In addition to the abovementioned set of cyanoacetamides, 2-(*N,N*-dimethylamino)-*N*-hydroxy-2-oxoacetimidoyl cyanide (DmOx, **9**) was also evaluated. This molecule differs from AmOx (**7**) in its capacity to act as a hydrogen donor and in its nonplanar structure (Figure 2).[22] While oximes **5**–**9** have been broadly applied in organometallic chemistry, biomedicine and agrochemistry, to our knowledge, the only reports of an additive to carbodiimides presenting an *N*-alkyl-cyanoacetamido oxime scaffold were described in the 1970s, excluding the recent reevaluation of Oxyma (**3**).[17, 22–25] At the beginning of that decade, Itoh determined the acidity of Oxyma (**3**, p*K*_a_ 4.6) and AmOx (**7**, p*K*_a_ 5.2), and evaluated the racemization suppression of the former in a dipeptide model.[22] A few years later, Izdebski further examined the capacity of Oxyma (**3**) and AmOx (**7**), together with other less suitable oximes, to decrease epimerization in *N*,*N′*-dicyclohexylcarbodiimide (DCC)-mediated couplings using an optimized protocol.[24, 25] Nonetheless, given the preliminary character of these studies, they did not provide a conclusive overview of the performance of these oximes, although a lower reactivity of acetamido analogue **7** was envisaged.[22, 25] Here, we revised the capacity of AmOx (**7**) together with *N*-alkyl-cyanoacetamido oximes **5**–**9** to retain the optical configuration of the activated amino acid and their coupling efficiency, while comparing their performance with that of HOSu (**4**).

## Results and Discussion

### Synthetic approach

The pool of *N*-alkyl-cyanoacetamido oximes selected for screening as additives (**5**–**9**) included several linear and cyclic *N*-alkyl moieties, which involve diverse bulkiness and the possibility to allow hydrogen-bonding interactions. In comparison with the ester functionality within Oxyma (**3**), the carboxamido group displays restricted conformational rotation around the amide bond, as shown earlier by 1H NMR studies.[22], 23b, f This limitation could have some impact on the reactivity of the corresponding esters.

**Scheme 1:**
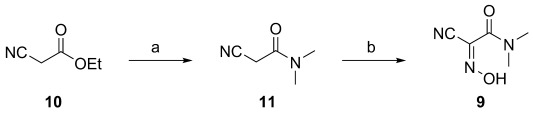
Two-step synthetic route to cyanooxime, DmOx (9). *Reagents and conditions:* a) ethyl cyanoacetate (10), aq Me_2_NH (40 %), 70 °C; b) Intermediate 11, NaNO_2_, MeOH/H_2_O, 2 h at RT.

Regarding the syntheses of the proposed oximes, **5**–**8** were already reported in the evaluation of the derived Fmoc-carbonates.[21] Following the same methodology, DmOx (**9**) was synthesized in high yield from ethyl cyanoacetate (**10**) in only two steps by means of an amidation/nitrosation sequence (Scheme 1).[21] In the first step of this synthetic approach, ethyl cyanoacetate (**10**) was readily transformed into *N,N*-dimethylcyanoacetamide (**11**) after reaction with dimethylamine at 70 °C. Next, the subsequent Meyer nitrosation of intermediate **11** in a methanolic solution of sodium nitrite afforded desired oxime **9** in 76 % overall yield.[26] Although this reaction sequence leading to DmOx (**9**) has already been described in the literature, our optimized methodology leads to a significant improvement in yield and purity compared with former strategies, which describe an initial amidation in aqueous medium or do not generate methyl nitrite in situ.[23] Like cyanoacetamido oximes **5**–**8**, the high water solubility of DmOx (**9**) had a positive impact on the final aqueous work-up.[21]

### Control of epimerization

**Figure 3:**
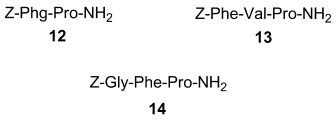
Peptide models used to test the reduction of epimerization induced by *N*-alkyl-cyanoacetamido oximes.

Retention of the optical configuration during amino acid coupling is central to peptide synthesis, especially when manufacturing active principle ingredients (APIs).[9, 27] The undesired loss of chirality at C_α_ is dramatic in more sensitive building blocks, such as glycopeptides.[28] In order to study the impact of epimerization when using *N*-alkyl-cyanoacetamido oximes **5**–**9** in solution phase, we selected peptides **12**–**14** as test models, which are a reliable platform to study the loss of configuration during stepwise and [2+1] fragment couplings (Figure 3).[29] After *N*,*N′*-diisopropylcarbodiimide (DIC)-mediated coupling of equimolar amounts of acid and amine and aqueous work-up, the impact of epimerization on peptides **12**–**14** was determined by reversed-phase HPLC, using optimized conditions that achieve clear separation of the two epimers. Simultaneously, a direct comparison with HOSu (**4**) and Oxyma (**3**), the latter as the most efficient additive, was established in every model.

The performances of additives **3**–**9** in a stepwise coupling was examined during the coupling of *N*-carboxybenzyl (Cbz)-protected Z-l-phenylglycine (Z-Phg-OH) onto l-prolinamide (H-Pro-NH_2_), resulting in dipeptide **12** (Table 1).29a The α-phenyl moiety in Phg ensured a high sensitivity towards epimerization, because of the pronounced stability of the resulting anion. In this model, HOSu (Entry 2, Table 1) rendered the lowest yield of dipeptide and an unacceptable loss of configuration. The percentage of epimerization achieved (32.7 %) was even more detrimental, taking into account that the absence of preactivation in a peptide coupling usually attenuates epimerization. Remarkably, all *N*-alkyl-cyanoacetamido oximes **5**–**9** surpassed HOSu (**4**) in their capacity to maintain the desired chirality of the test dipeptide (cf. Entries 2 and 3–7, Table 1). In particular, PipOx (**6**) was the least efficient in reducing epimerization, but still afforded three times higher purity than HOSu (**4**) (cf. Entry 2 and 4, Table 1). In comparison with MorOx (**5**), the presence of a proton-accepting oxygen in the six-membered ring caused a considerable increase in purity of the desired peptide (cf. Entry 3 and 4, Table 1).[30] Noteworthy, AmOx (**7**) and *N*-Oxyma (**8**) showed similar configuration retentions to Oxyma (**3**), which afforded only 1.0 % of dl epimer, with a simultaneous improvement in yield (cf. Entry 1 and 5, 6, Table 1).[17]

**Table 1:** Yield and epimerization during the formation of Z-Phg-Pro-NH_2_ (12) using additives 3–9 (solution phase synthesis)[a]

Entry	Coupling Reagent	Yield [%]	dl [%][b]
1	DIC/Oxyma (**3**)	89.9[c]	1.0[c]
2	DIC/HOSu (**4**)	70.3	32.7
3	DIC/MorOx (**5**)	88.4	5.7
4	DIC/PipOx (**6**)	86.8	10.7
5	DIC/AmOx (**7**)	81.4	3.0
6	DIC/*N*-Oxyma (**8**)	92.1	3.8
7	DIC/DmOx (**9**)	93.4	8.3

[a] Couplings were performed without pre-activation in DMF at RT.

[b] The ll and dl epimers of this peptide model have been described in the literature.29a Retention times (*t*_R_) for each epimer were identified after co-injection with a pure ll sample.

[c] Results are extracted from Ref. [17].

Once the stepwise system was evaluated, the next step in the epimerization study of *N*-alkyl-cyanoacetamido oximes **5–9** corresponded to the [2+1] assembly of dipeptide Z-Phe-Val-OH onto H-Pro-NH_2_, affording tripeptide **13** (Table 2).29b The activation of a dipeptide acid is a very interesting scenario to test the performance of an additive, since oxazolone formation is promoted as a result of the electron-donating effect of the *N*-aminoacyl substitution.[17, 30] As observed in Table 2, the percentages of unwanted epimer were considerably higher than in the previous stepwise system. Following the trend observed in the synthesis of Z-Phg-Pro-NH_2_ (**12**), most of the cyanoacetamido oximes induced an approximately twofold higher retention of configuration than HOSu (**4**). Nonetheless, this [2+1] system produced moderate yields (66–84 %).

**Table 2:** Yield and epimerization during the [2+1] formation of Z-Phe-Val-Pro-NH2 (13) using additives 3–9 (solution phase synthesis).[a]

Entry	Coupling Reagent	Yield [%]	ldl [%][b]
1	DIC/Oxyma (**3**)	89.8	12.8
2	DIC/HOSu (**4**)	73.1	24.7
3	DIC/MorOx (**5**)	80.5	13.2
4	DIC/PipOx (**6**)	71.5	32.6
5	DIC/AmOx (**7**)	66.7	14.8
6	DIC/*N*-Oxyma (**8**)	83.6	18.4
7	DIC/DmOx (**9**)	79.5	17.0

[a] Couplings were performed without pre-activation in DMF at RT.

[b] The lll and ldl epimers of this tripeptide model have been described in the literature.29b Retention times (*t*_R_) for each epimer were identified after co-injection with a pure sample.

Concerning the relative performance of the *N*-alkyl-cyanoacetamido oxime series (**5**–**9**), PipOx (**6**) again showed the least efficient control of optical purity, providing an even higher percentage of ldl epimer than HOSu in this system (cf. Entry 2 and 4, Table 2). In sharp contrast, its morpholino analogue **5** showed a similar level of performance to Oxyma (**3**), providing an almost threefold higher retention of chirality than PipOx (**6**) (cf. Entries 1 and 3, 4, Table 2). Moreover, MorOx (**5**) was one of the most efficient oximes in terms of yield of target tripeptide **13**. With regard to AmOx (**7**), a remarkably low content of epimer was detected, although accompanied by one of the lowest yields in the oxime series (Entry 5, Table 2). Finally, *N*-Oxyma (**8**) was again clearly less efficient than its parent Oxyma (**3**) at preventing the loss of chirality (Entry 6, Table 2).

The last epimerization model used to evaluate cyanoacetamido oximes **5–9** also consisted of a [2+1] segment coupling, in this case rendering tripeptide Z-Gly-Phe-Pro-NH_2_ (**14**) (Table 3).29b Although this system is undoubtedly less sensitive than the previous one (**13**), valuable data were gathered. Unlike the synthesis of Z-Phe-Val-Pro-NH_2_ (**13**), PipOx (**6**) was not only more efficient than HOSu in decreasing epimerization (cf. Entry 2 and 4, Table 3), but also superseded the performance of morpholine derivative **5** (cf. Entry 3 and 4, Table 3). Consistent with previous epimerization models, the performance of HOSu (**4**) was poorer than *N*-alkyl-cyanoacetamido oximes **5**–**9**, including the relative yields obtained (cf. Entry 2 and 3–7). Surprisingly, MorOx (**5**) afforded one of the highest obtained contents of ldl epimer, along with *N*-Oxyma (**8**), which offers threefold less pure crude products than Oxyma (**3**) (cf. Entries 1 and 3, 6, Table 3). Finally, of the cyanoacetamido oximes tested in the stepwise and fragment couplings **12**–**14**, AmOx (**7**) regularly performed at a remarkable level, offering the closest percentages of epimer to those obtained with Oxyma (**3**). Thus, AmOX (**7**) stands out as one of the most promising derivatives to substitute HOSu (**4**) as an epimerization-suppressant additive.

**Table 3:** Yield and epimerization during the [2+1] formation of Z-Gly-Phe-Pro-NH_2_ (14) using additives 3–9 (solution phase synthesis)[a]

Entry	Coupling Reagent	Yield [%]	ldl [%]
1	DIC/Oxyma (**3**)	90.2	2.7
2	DIC/HOSu (**4**)	80.4	9.0
3	DIC/MorOx (**5**)	88.3	8.0
4	DIC/PipOx (**6**)	86.6	7.6
5	DIC/AmOx (**7**)	88.9	4.3
6	DIC/*N*-Oxyma (**8**)	87.6	8.3
7	DIC/DmOx (**9**)	87.9	5.4

[a] Couplings were conducted without pre-activation in DMF at RT.

[b] The lll and ldl epimers of this tripeptide model have been described in the literature.29b Retention times (*t*_R_) for each epimer were identified after co-injection with a pure sample.

### Coupling potency

**Figure 4:**
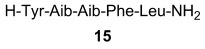
Aib-enkephaline pentapeptide (15) used as coupling efficiency model to test *N*-alkyl-cyanoacetamido oximes 5–9.

A key step in the assessment of the quality of cyanoacetamido oximes **5**–**9** as additives to carbodiimides was the evaluation of their acylation potency in hindered couplings. To emphasize differences in the reactivity of the various active esters, peptide models containing demanding *N*-methyl or α,α-dialkyl amino acid junctions are often used.[17, 30] Among these, those featuring two or more consecutive α-aminoisobutyric acid (Aib) residues are particularly difficult to achieve, not only because of their considerable bulkiness, but also due to the stabilization of helical structures by the Thorpe–Ingold effect.[31] Hence, additives **3**–**9** were used in the solid-phase assembly of the Aib-enkephaline pentapeptide (**15**, Figure 4).[17, 30, 32]

The demanding conditions of this efficiency model (using short coupling times) were responsible for the very high percentage of des-Aib deletion tetrapeptide H-(Tyr-Aib-Phe-Leu-NH_2_), obtained with the various additives (**3**–**9**; Table 4). Consistent with the trend observed in the epimerization experiments, the coupling performance of HOSu (**4**) was surpassed by all *N*-alkyl-cyanoacetamido oximes **5**–**9** (cf. Entry 2 and 3–7, Table 4). Thus, most of the crude mixture obtained when using HOSu (**4**) consisted of deletion tripeptides, and no desired pentapeptide **15** was detected (Entry 2, Table 4). In contrast, oximes **5–9** produced 10–30 % of Aib-pentapeptide **15** (Entries **3**–**7**, Table 4). Particularly impressive was the efficiency displayed by the DIC/DmOx (**9**) system, which yielded the highest percentage of pentapeptide **15** in the cyanoacetamido oxime series (Entry 7, Table 4), an efficiency comparable to HOAt (**2**) in similar conditions and similar to that of Oxyma (**3**, Entry 1, Table 4).[29] The Aib-enkephaline model (**15**) also highlighted the enormous difference in acylation potency between DmOx (**9**) and the rest of the cyanoacetamido oximes (Entry 7 and 3–6, Table 4). In a similar fashion to the epimerization assessment, PipOx (**6**) was found to be the least potent additive in amino acid activation (Entry 4, Table 4). With respect to the acetamido analogue of Oxyma (**8**), a threefold decrease in reactivity was observed in comparison with parent ester **3**.

**Table 4:** Percentage of tetrapeptide des-Aib (H-Tyr-Aib-Phe-Leu-NH_2_) during solid-phase assembly of pentapeptide 15 (H-Tyr-Aib-Aib-Phe-Leu-NH_2_)[a]

Entry	Coupling Reagent	**15** [%]	des-Aib [%][b]
1	DIC/Oxyma (**3**)[c]	46.5	53.5
2	DIC/HOSu (**4**)	0	12.2
3	DIC/MorOx (**5**)	13.6	75.8
4	DIC/PipOx (**6**)	10.9	78.0
5	DIC/AmOx (**7**)	18.9	73.1
6	DIC/*N*-Oxyma (**8**)	15.4	79.9
7	DIC/DmOx (**9**)	31.3	68.7

[a] 10 min pre-activation and 30 min coupling times were generally applied, except for Aib-Aib (30 min double coupling).

[b] Deletion tetrapeptide des-Aib was identified by peak overlap in reversed-phase HPLC with an authentic sample obtained in solid-phase.

[c] Results are taken from Ref. [17].

## Conclusion

Here, we evaluated several *N*-alkyl-cyanoacetamido oximes (**5**–**9**) as additives to carbodiimides in substitution of HOSu (**4**) for the construction of stable amino acid esters. One of the main advantages of the proposed cyanoacetamido oximes, which can be accessed by a shown optimized amidation/nitrosation procedure, is their enhanced water solubility, which was particularly impressive in MorOx (**4**). Consequently, not only was byproduct removal increased, thereby allowing more facile work-up, but the water-soluble nature of cyanoacetamido oximes also allows their use in protein conjugation and labeling in addition to peptide synthesis.

A thorough assessment of the reduction of epimerization in various stepwise and [2+1] fragment models and the coupling efficiency in demanding junctions indicated that the cyanoacetamido family of additives showed a much greater capacity of configuration retention and acylation potency than *N*-hydroxysuccinimide (**4**). Among the *N*-alkyl-cyanoacetamido oximes **5–9**, PipOx (**6**) can be considered the least efficient analogue of the series. Nonetheless, the inclusion of an oxygen atom in the six-membered ring (MorOx, **5**) not only resulted in an increase in solubility but also in coupling efficiency. With regard to *N*-Oxyma (**8**), the envisaged decrease in reactivity and control of configuration by abandoning the ester moiety in Oxyma (**3**) was confirmed. Last but not least, carboxamido analogues AmOx (**7**) and DmOx (**9**) are highlighted as the most promising cyanoacetamido oxime derivatives. While the unsubstituted carboxamide (**7**) stands out in the epimerization experiments due to its marked capacity to retain chirality, DmOx (**9**) showed superiority to the rest of the oximes in the reactivity of the corresponding active ester.

## Experimental Section

### Instruments and materials

All solvents used for recrystallization, extraction, HPLC analyses and TLC were of HPLC-reagent grade. They were distilled before use, and stored under dry conditions. 1 H NMR were recorded on a Mercury 400 MHz spectrometer (Varian) at RT, using tetramethylsilane (TMS) as internal standard. Chemical shifts were reported in *δ* units (parts per million) relative to TMS. Melting points were determined using a Mel-Temp apparatus (B-540, Büchi) and are uncorrected. Reaction monitoring and determination of the purity of the compounds were performed by TLC on silica gel-protected aluminium sheets (Type 60 GF254, Merck), and the spots were detected by exposure to UV light at *λ*=254 nm. Analytical separation and characterization was conducted on a reversed-phase 2695 HPLC separation module (Waters) using a SunFire C_18_ column (3.5 μm, 4.6×100 mm) and a linear gradient of CH_3_CN in H_2_O supplemented with 0.1 % trifluoroacetic acid (TFA) at a flow rate of 1.0 mL min^−1^. Detection at 220 nm was performed using a coupled 2998 PDA UV detector (Waters). Chromatograms were processed with Empower software (Pro version). Peptide mass was detected by means of an HPLC-PDA system as described above, coupled to a Micromass ZQ mass detector (Waters), using the MassLynx 4.1 software. The compounds were named using ChemDraw Ultra version 11 (CambridgeSoft Corporation).

### Preparation of N*-*alkyl-cyanoacetamido oximes[21]

Oximes **5**–**8** were synthesized in a previous related publication addressing their inclusion in Fmoc-carbonates and were used for the present study.[21] The procedure used is an optimization of earlier amidation/nitrosation protocols and is detailed below for the preparation of DmOx (**9**).[23]

**Preparation of 2-(dimethylamino)-*N*-hydroxy-2-oxoacetimidoyl cyanide (DmOx, 9)**:[21, 23] An aq solution of Me_2_NH (40 %, 40 mL, 350 mmol) was added to ethyl cyanoacetate (**10**, 22.6 g, 200 mmol) at −10 °C. The reaction mixture was stirred for 1 h at RT and then refluxed for 6 h at 70 °C. Next, the solvent was evaporated in vacuo, leaving 2-cyano-*N,N*-dimethylacetamide (**11**) as an oil, which was used in the next nitrosation step without further purification. Gaseous methyl nitrite was generated from a suspension of NaNO_2_ (8.3 g) in a mixture of MeOH (10 mL) and H_2_O (10 mL) by dropwise addition of a mixture of concd H_2_SO_4_ (5 mL) and H_2_O (10 mL) and introduced to an ice-cooled solution of intermediate **11** (100 mmol) and NaOH (4 g, 100 mmol) in MeOH (30 mL). The mixture was stirred for 2 h at RT and concentrated to dryness in vacuo. The residue was dissolved in the least amount of H_2_O (5 mL), ice-cooled, and acidified with concd HCl to induce precipitation. The product was obtained as white crystals (21.43 mg, 76 %)[23]: mp 138 °C; ^1^H NMR (C_3_D_6_O): *δ*=3.02, 3.18 (2s, 6 H, C*H*_3_), 13.09 ppm (s, 1 H, O*H*, D_2_O exchangeable).

### General method for racemization experiments[17, 30]

Acid Z-Phg-OH, Z-Phe-Val-OH, or Z-Gly-Phe-OH (0.125 mmol), DIC (20 μL, 0.125 mmol), and additive (0.125 mmol) were dissolved in dimethylformamide (DMF; 2 mL) at 0 °C. H-Pro-NH_2_ (14.3 mg, 0.125 mmol) was added, and the reaction mixture was stirred for 1 h at 0 °C and overnight at RT. The reaction mixture was diluted with EtOAc (25 mL), extracted with 1 n HCl (2×5 mL), 1 n NaHCO_3_ (2×5 mL), and saturated NaCl (2×5 mL), dried over anhyd MgSO_4_ and filtered. The solvent was removed in vacuo, and the crude peptide was directly analyzed by reversed-phase HPLC.

**Z-Phg-Pro-NH_2_**: A linear gradient of 25–50 % CH_3_CN in H_2_O supplemented with 0.1 % TFA over 25 min was applied: *t_R_*(ll)=9.3 min, *t_R_* (dl)=9.9 min.

**Z-Phe-Val-Pro-NH_2_**: A linear gradient of 20–70 % CH_3_CN in H_2_O supplemented with 0.1 % TFA over 14 min was applied: *t_R_*(lll)=6.7 min, *t_R_*(ldl)=7.1 min.

**Z-Gly-Phe-Pro-NH_2_**: A linear gradient of 20–70 % CH_3_CN in H_2_O supplemented with 0.1 % TFA over 14 min was applied: *t_R_*(lll)=5.5 min, *t_R_*(ldl)=5.8 min.

### Solid phase synthesis of H-Tyr-Aib-Aib-Phe-Leu-NH_2_ (15)

Pentapeptide **15** was prepared as reported.[17, 30] Fmoc-RinkAmide-PS resin (0.7 mmol g^−1^, 100 mg) was deblocked with piperidine in DMF (20 %, 10 mL) for 10 min, and washed with DMF (2×10 mL), CH_2_Cl_2_ (2×10 mL), and DMF (2×10 mL). Fmoc-Leu-OH (3 equiv), DIC (3 equiv) and additive (3 equiv) were mixed in DMF (0.5 mL), preactivated for 10 min and then added to the resin. The resulting slurry was stirred slowly for 1 min and allowed to couple for 30 min (double coupling for Aib-Aib). The ninhydrin test was performed during the introduction of the first residue (negative). The resin was subsequently washed with DMF (2×10 mL) and Fmoc removed with piperidine in DMF (20 %, 10 mL) for 7 min. Next, the peptide resin was washed with DMF (2×10 mL), DCM (2×10 mL), and DMF (2×10 mL), and coupled with the next amino acid. Coupling and deblocking steps were repeated to provide the pentapeptide. The percentage of des-Aib (Tyr-Aib-Phe-Leu-NH_2_) during solid-phase assembly of pentapeptide **15** was confirmed by peak overlap in the presence of an authentic sample. Crude pentapeptide **15** was analyzed by reversed-phase HPLC. A linear gradient of 10–90 % CH_3_CN in H_2_O supplemented with 0.1 % TFA over 20 min was applied: *t_R_* (**15**)=7.30 min, [*M*+H]^+^=611.35; *t_R_* (des-Aib)=7.50 min, [*M*+H]^+^=526.30.

## References

[1] El-Faham A, Albericio F (2011). Chem. Rev.

[2] Valeur E, Bradley M (2009). Chem. Soc. Rev.

[3] Han S-Y, Kim Y-A (2004). Tetrahedron.

[4] Humphrey JM, Chamberlin AR (1997). Chem. Rev.

[5] Weisz I, Roboz J, Bekesi G (1996). Tetrahedron Lett.

[6] Carpino LA, Beyermann M, Wenschuh H, Bienert M (1996). Acc. Chem. Res.

[7] Senokuchi K, Nakai H, Nagao Y, Sakai Y, Katsube N, Kawamura M (1998). Bioorg. Med. Chem.

[8] Albericio F (2000). Biopolymers.

[9] Carpino LA, Ghao HG, Beyermann M, Bienert M (1991). J. Org. Chem.

[10] Bodánszky M (1955). Nature.

[11] Subiros-Funosas R, El-Faham A, Albericio F, Rappoport Z, Liebman JF (2011). N-hydroxylamines for Peptide Synthesis, The Chemistry of Hydroxylamines, Oximes and Hydroxamic Acids, Vol. 2(2).

[12] Carpino LA, El-Faham A (1999). Tetrahedron.

[13] Hachmann J, Lebl M (2006). Biopolymers.

[14a] König W, Geiger R (1970). Chem. Ber.

[14b] König W, Geiger R (1970). Chem. Ber.

[15] Carpino LA (1993). J. Am. Chem. Soc.

[16] Wehrstedt KD, Wandrey PA, Heitkamp D (2005). J. Hazard. Mater.

[17] Subirós-Funosas R, Prohens R, Barbas R, El-Faham A, Albericio F (2009). Chem. Eur. J.

[18] Anderson GW, Zimmerman JE, Callahan FM (1964). J. Am. Chem. Soc.

[19] Meng QH, Yu MJ, Zhang HF, Ren JC, Huang DY (2007). Dyes Pigm.

[20a] Sigler GF, Fuller WD, Chaturvedi CN, Goodman M, Verlander M (1983). Biopolymers.

[20b] Milton RC, Becker E, Milton SC, Baxter JEJ, Elsworth JF (1987). Int. J. Pept. Protein Res.

[21] Khattab SN, Subirós-Funosas R, El-Faham A, Albericio F (2012). Tetrahedron.

[22] Itoh M (1973). Bull. Chem. Soc. Jpn.

[23a] Gerasimchuk N, Gamian A, Glover G, Szponar B (2010). Inorg. Chem.

[23b] Ciba-Geigy A-G (1980).

[23c] Basheer A, Yamataka H, Ammal SC, Rappoport Z (2007). J. Org. Chem.

[23d] Eddings D, Barnes C, Gerasimchuk N, Durham P, Domasevich K (2004). Inorg. Chem.

[23e] Gerasimchuk N, Maher T, Durham P, Domasevitch KV, Wilking J, Mokhir A (2007). Inorg. Chem.

[23f] Domasevitch KV, Gerasimchuk NN, Mokhir A (2000). Inorg. Chem.

[24] Izdebski J (1975). Roczniki Chemii.

[25] Izdebski J (1979). Pol. J. Chem.

[26] Meyer V (1873). Ber. Dtsch. Chem. Ges.

[27] Davies JS, Mohammed AK (1981). J. Chem. Soc. Perkin Trans. 1.

[28] Zhang Y, Muthana SM, Farnsworth D, Ludek O, Adams K, Barchi JJ, Gildersleeve JC (2012). J. Am. Chem. Soc.

[29a] Wenschuh H, Beyermann M, Haber H, Seydel JK, Krause E, Bienert M (1995). J. Org. Chem.

[29b] Carpino LA, El-Faham A, Albericio F (1995). J. Org. Chem.

[30a] El-Faham A, Albericio F (2008). J. Org. Chem.

[30b] El-Faham A, Subirós-Funosas R, Prohens R, Albericio F (2009). Chem. Eur. J.

[31a] Toniolo C, Bonora GM, Bavoso A, Benedetti E, Di Blasio B, Pavone V, Pedone C (1983). Biopolymers.

[31b] Jastrzabek KG, Subiros-Funosas R, Albericio F, Kolesinska B, Kaminski ZJ (2011). J. Org. Chem.

[31c] Jung ME, Piizzi G (2005). Chem. Rev.

[32a] Carpino LA, El-Faham A, Minor CA, Albericio F (1994). J. Chem. Soc. Chem. Commun.

[32b] Auvin-Guette C, Frerot E, Coste J, Rebuffat S, Jouin P, Bodo B (1993). Tetrahedron Lett.

